# Adherence to 24-h Movement Guidelines and Depressive Status During the Coronavirus Disease Outbreak: A Cross-Sectional Japanese Survey

**DOI:** 10.3389/ijph.2023.1604647

**Published:** 2023-02-24

**Authors:** Takahisa Ohta, Madoka Ogawa, Naoki Kikuchi, Hiroyuki Sasai, Takanobu Okamoto

**Affiliations:** ^1^ Research Institute for Sport Science, Nippon Sport Science University, Tokyo, Japan; ^2^ Research Team for Promoting Independence and Mental Health, Tokyo Metropolitan Institute for Geriatrics and Gerontology, Tokyo, Japan

**Keywords:** sedentary behavior, physical activity, sleep duration, depression, 24-h movement guidelines

## Abstract

**Objectives:** The coronavirus disease 2019 (COVID-19) pandemic has affected people’s physical activity, sedentary behavior, and sleep. This study aimed to clarify the association between combining these factors, integrated as adherence to 24-h movement guidelines, and depressive status during the COVID-19 pandemic.

**Methods:** At the end of October 2020, we sent self-administered questionnaires to 1,711 adults aged ≥18. We assessed physical activity, sedentary behavior, sleep duration, adherence to 24-h movement guidelines, depressive status, and confounding factors.

**Results:** Of the 640 valid responses, 90 (14.1%) reported a depressive status. Multivariable odds ratios (95% confidence interval) of depressive status were 0.22 (0.07, 0.71) for all three recommendations of the 24-h movement guidelines and those who met none of the recommendations as reference. The number of guidelines met was associated with depressive status in a dose-response fashion.

**Conclusion:** Meeting the 24-h movement guidelines was associated with a lower prevalence of depressive status during the COVID-19 pandemic. Adults should adhere to these guidelines to maintain their mental health during future quarantine life.

## Introduction

The coronavirus disease 2019 (COVID-19) outbreak has drastically affected the economy and people’s lifestyles worldwide [1, 2] Physical activity (PA) decreased during the pandemic due to implementing a quarantined lifestyle [3] Sadly, behavioral changes such as decreased PA and prolonged sedentary behavior (SB) may increase the risk of adverse health outcomes, including cancer, cardiovascular disease, diabetes, and depression [4, 5]. The prevalence of depression during the COVID-19 pandemic was reportedly 27%–45% worldwide and has become a serious public health concern [6–11].

Depression is a common illness in Japan and a proven risk factor for suicide; it also brings a substantial economic burden [12, 13] Therefore, preventive measures are necessary to avoid the onset of depression and promote mental health. Previous studies before the COVID-19 pandemic suggested that moderate PA slows down the onset of depression. A systematic review and meta-analysis showed a non-linear dose-response association between PA and health outcomes, with higher PA preventing the onset of depression compared with lower PA [14]. A longer time spent in SB also carried a higher risk of depression, with a relative risk of 1.25 (95% confidence interval [CI]: 1.16–1.35) for a longer versus a shorter time spent in SB [15].

A rapid systematic review during the pandemic demonstrated that a higher volume and frequency of moderate-to-vigorous physical activity (MVPA) was associated with a 12%–32% and 15%–34% lower prevalence of depression and anxiety, respectively [16]. Other studies showed that low PA was also associated with mental illness during the COVID-19 pandemic [17, 18]. However, these studies had notable limitations and challenges.

First, the above rapid systematic review focused only on individual components such as PA, which may have unknown confounding factors. An individual’s 24-h daily activity comprises three components, PA, SB, and sleep duration. These three components are codependent rather than independent, and seeing them as a single frame is beginning to take hold as the latest public health concept. The World Health Organization (WHO) and academic societies from several countries strongly support this concept [17–19] In particular, a 24-h movement guideline of the Canadian Society of Exercise Physiology recommends limiting SB to <8 h per day, MVPA to at least 150 min per week, and 7–9 h of sleep (7–8 h for adults ≥65 years) [17]. Furthermore, few studies have examined the association between mental health and 24-h movement guidelines, including depression, although associations with adiposity markers and cardiovascular markers have been reported [20].

Second, there is a lack of information on the associations between mental health and 24-h movement for the Japanese population during the COVID-19 pandemic. The policies (e.g., lockdown) to prevent the spread of COVID-19 differ among countries. Since Japan declared a state of emergency with fewer legal restrictions than other countries, it is impossible to compare Japan with other countries. For example, the studies conducted during the lockdown showed a decrease in PA and an increase in SB among U.S. college students and adults compared to pre-lockdown conditions, and adverse mental health consequences were also observed [21, 22]. In Japan, an increase in suicide rates during the pandemic has been reported, and suggestions for preventive strategies to maintain mental wellbeing must be an urgent issue [23]. Thus, clarifying the importance of 24-h movement guidelines for each country’s response during a pandemic may be a piece of important fundamental knowledge for preparing for the next unknown infectious disease.

This study aimed to clarify the association between adherence to the 24-h movement guidelines (in terms of PA, SB, and sleep duration) and the prevalence of depressive status among middle-aged and older adults during the COVID-19 outbreak in Japan. The findings of this study will allow us to add to the knowledge of preventive medicine targeted toward avoiding depressive symptoms in current and future similar crises that may require implementing a quarantine lifestyle.

## Methods

### Design and Participants

This cross-sectional survey, a part of the Setagaya- Aoba study, was conducted at the end of October 2020 [24]. On either side of the survey period, states of emergency were declared in Japan due to the COVID-19 pandemic. The first state of emergency lasted from 7 April to 25 May 2020, and the second from 8 January to 21 March 2021. The Nippon Sport Science University is located in Setagaya, Tokyo, and Aoba, Yokohama-city has direct access to the metropolitan area and is called a commuter town. The Nippon Sport Science University holds physical fitness test events for citizens annually. These events involve approximately 1,000 persons living around the university each year and contribute their health assessments for free. Event guidance is provided on the bulletin board and the university website from September to October every year. After an online survey announcement, individuals who had participated in our fitness test events since 2017 received the mail-based questionnaire. Participants were excluded for the following reasons: age <18 years old, non-response, or response without physical activity or a depressive questionnaire.

### Physical Activity, SB, and Sleep Duration

Physical activity was assessed using the Global Physical Activity Questionnaire (GPAQ, Japanese version), which was developed initially by the WHO [25]. The weekly cut-off for physical activity among adults was based on the 24-h movement guidelines and defined as performing moderate-to-vigorous-intensity aerobic physical activity for >150 min per week. SB was also assessed using the GPAQ using the question, “How much time do you usually spend sitting or reclining on a typical day?” SB was evaluated based on the 24-h movement guidelines and categorized as < 8 h per day according to the duration. Sleep duration was assessed using a single self-administered question, “How long do you usually sleep a day?” Participants could respond by reporting the time in hours and minutes. Based on 24-h movement guidelines, the limits were set as 7–9 h per day for adults 18–64 years old and 7–8 h for adults ≥65 years old [17].

### Adherence to 24-h Movement Guidelines

Participants were categorized into eight groups to assess adherence to components of the Canadian 24-h movement guidelines as follows: 1) not meeting any recommendations, 2) meeting only SB recommendations, 3) meeting only MVPA recommendations, 4) meeting only sleep duration recommendations, 5) meeting SB and MVPA recommendations, 6) meeting SB and sleep duration recommendations, 7) meeting MVPA and sleep duration recommendations, and 8) meeting all recommendations of the 24-h movement guidelines. In addition, participants were categorized into the following four groups to assess their adherence to the number of guideline recommendations: 1) not meeting any recommendations, 2) meeting only one recommendation, 3) meeting two recommendations, and 4) meeting all recommendations.

### Depressive Status

The Center for Epidemiologic Studies Depression Scale (CES-D) was used to assess depressive status [26]. The CES-D is a valid and reliable tool comprising 20 items. The CES-D instrument evaluates whether the respondent has experienced depression symptoms, such as restless sleep, poor appetite, and feeling of loneliness, within the preceding week. Responses consisted of symptom frequency and ranged from 0 to 3 for each item. For example, the responses “rarely/never,” “sometimes,” “moderately/most of the time,” and “mostly/almost all the time” were scored as 0, 1, 2, and 3 points, respectively. We also summed the score of the 20 items (range: 0–60) and assigned a score of ≥16 as the cut-off for depressive states.

### Confounding Factors

Body mass index was calculated using self-reported height and weight. We used a self-administered questionnaire to obtain data regarding smoking status (yes/no), alcohol consumption (yes/no), changes in income in 2019 (compared with before the COVID-19 outbreak), living alone (yes/no), daily use of social networking service (SNS; yes/no), history of depression (yes/no), the number of chronic diseases requiring medicine, and menopause (yes/no).

### Statistical Analysis

To reduce the bias of missing values, we conducted multiple imputations using every explanatory variable and obtained 20 datasets. First, an analysis of each dataset was conducted each dataset, and then the results of 20 multiple imputation datasets were integrated into one result. The continuous variables are presented as means (standard deviations) or medians (interquartile ranges) based on the normality test results. The categorical variables were shown as numbers (percentages).

A logistic regression model was used to perform a univariate analysis between each confounding factor and depressive status. The logistic regression analyses examined the association between meeting the 24-h movement guideline and the prevalence of depressive status. The models were adjusted for age, body mass index, smoking habits [27], alcohol habits [28], changes in income since 2019, presence of a housemate, daily use of SNS [29] a history of depression, number of chronic diseases requiring medicine [30], and menopause [31]. The multivariable odds ratios (ORs) and 95% CIs for the associations between meeting the 24-h movement guidelines and depressive status were calculated using the “not meeting the guidelines” group as a reference. A continuous variable (the number of guidelines met) was included in a separate model to evaluate the linear associations. All the statistical analyses were performed using the SPSS version 25 (IBM, Inc., Chicago, IL, United States). A *p-*value of <0.05 was considered to be statistically significant.

## Results

During 2017–2019, 1,711 adults participated in physical fitness tests at the Tokyo Setagaya and Yokohama Kenshidai (Aoba-ward) Nippon Sport Science University campuses. All these individuals were eligible to participate in the study. Of the 640 valid responders, 90 (14.1%) reported a depressive status (Table 1).

**TABLE 1:** Characteristics of participants (Setagaya- Aoba study, Japan, 2020).

	Overall	Number of guideline meeting			
		0	1	2	3
	*n* = 640	*n* = 38	*n* = 166	*n* = 293	*n* = 143
No. of cases, n (%)	90 (14.1)	12	27	38	13
CES-D score	9.4 (6.7)	13.2 (8.0)	9.7 (7.4)	9.1 (6.1)	8.6 (6.3)
Sex					
Men, n (%)	268 (41.7)	15	73	119	61
Women, n (%)	372 (58.3)	23	93	174	82
Age, year	64.1 (14.0)	60.9 (13.1)	60.7 (14.0)	65.2 (13.6)	66.5 (14.3)
Body mass index, kg/m^2^	21.8 (3.4)	22.3 (3.9)	21.7 (3.7)	22.0 (3.3)	21.6 (3.0)
Missing, n (%)	7 (1.1)	—	3 (1.8)	4 (1.4)	-
Smoking, n (%)	30 (4.7)	5 (13.2)	7 (4.2)	11 (3.8)	7 (4.9)
Missing, n (%)	9 (1.4)	—	6 (3.6)	3 (1.0)	-
Alcohol, n (%)	304 (47.3)	17 (44.7)	76 (45.8)	140 (47.8)	71 (49.7)
Missing, n (%)	9 (1.4)	—	5 (3.0)	3 (1.0)	1 (0.7)
Sleep duration, hr					
<6 h, n (%)	124 (19.4)	16 (42.1)	44 (26.5)	64 (21.8)	—
≤6, <8, n (%)	431 (67.3)	21 (55.3)	114 (68.7)	197 (67.2)	99 (69.2)
8 ≤, n (%)	85 (13.3)	1 (2.6)	8 (4.8)	32 (10.9)	44 (30.8)
Changes in income, n (%)					
Decreased	79 (14.8)	3 (7.9)	18 (10.8)	40 (13.7)	18 (12.6)
Unchanged	440 (68.8)	29 (76.3)	115 (69.3)	119 (40.6)	97 (67.8)
Increased	14 (2.2)	3 (7.9)	4 (2.4)	5 (1.7)	2 (1.4)
Missing, n (%)	107 (16.9)	3 (7.9)	29 (17.5)	49 (16.7)	26 (18.2)
Using SNS, n (%)	410 (36.3)	23 (60.5)	109 (65.7)	185 (63.1)	93 (65.0)
Living alone, n (%)	75 (12.1)	4 (10.5)	28 (16.9)	30 (10.2)	13 (9.1)
Missing, n (%)	10 (1.6)	—	4 (2.4)	5 (1.7)	1 (0.7)
History of depression, (%)	13 (2.0)	1 (2.6)	4 (2.4)	6 (2.0)	2 (1.4)
Number of chronic diseases, n (%)	—				
0	387 (60.5)	25 (65.8)	112 (67.5)	160 (54.6)	90 (62.9)
1	175 (27.3)	9 (23.7)	42 (25.3)	84 (28.7)	40 (28.0)
2	55 (8.6)	2 (5.3)	8 (4.8)	36 (12.3)	9 (6.3)
≥3	23 (3.6)	2 (5.3)	4 (2.4)	13 (4.4)	4 (2.8)
Menopause (women only)	290 (77.9)	17 (73.9)	70 (75.3)	142 (81.6)	61 (74.4)

Data were shown as mean (standard deviation) or number (percent).

CES-D, the Centers for Epidemiologic Studies Depression Scale; SNS, social networking service; SB, sedentary behavior; MVPA, moderate-to-vigorous physical activity.

Table 2 shows the association between the prevalence of depressive status and each variable. Age and the existence of housemates were associated with the prevalence of depressive status.

**TABLE 2:** Depressive status according to confounding factors (Setagaya- Aoba study, Japan, 2020).

Variables	No.	No. of cases	Prevalence^a^	OR (95% CIs)
Sex				
Women	372	62	166.7	1.00 (References)
Men	268	28	104.5	0.58 (0.36, 0.94)
Age^b^	640	90	140.6	0.82 (0.70, 0.96)
Body mass index	633	90	142.2	1.05 (0.98, 1.13)
Smoking				
No	601	82	136.4	1.00 (References)
Yes	30	6	200.0	1.73 (0.71, 4.22)
Alcohol				
No	327	46	140.7	1.00 (References)
Yes	304	41	134.9	0.94 (0.60, 1.48)
Sleep duration				
<6 h	124	25	201.6	1.69 (1.00, 2.85)
6 ≤, <8	431	56	129.9	1.00 (References)
8 ≤	85	9	105.9	0.79 (0.38, 1.67)
Changes in income				
Decreased	79	20	253.2	1.00 (References)
Unchanged	440	52	118.2	0.48 (0.27, 0.84)
Increased	14	3	214.3	0.58 (0.19, 1.82)
Using SNS				
No	230	33	143.5	1.00 (References)
Yes	410	57	139.0	0.96 (0.61, 1.53)
Living alone				
No	555	72	129.7	1.00 (References)
Yes	75	16	213.3	1.87 (1.03, 3.41)
History of depression				
No	627	88	140.4	1.00 (References)
Yes	13	2	153.8	1.11 (0.24, 5.11)
Medication of for chronic disease				
0	387	45	116.3	1.00 (References)
1	175	34	194.3	1.83 (1.12, 2.98)
2	55	9	163.6	1.49 (0.68, 3.24)
≥3	23	2	87.0	0.72 (0.16, 3.19)
Menopause (women only)				
No	73	17	232.9	1.00 (References)
Yes	290	43	148.3	0.57 (0.31, 1.08)

95%CI; 95% confidence interval, SNS; social networking service.

^a^ Prevalence ratio per 1000-person.

^b^ Per 10 years.

The association between meeting the 24-h movement guidelines and depressive status is shown in Table 3. After adjusting for various confounding factors and using “not meeting the guidelines” as a reference, participants who complained about MVPA, sleep duration independently or in combination with sleep duration, and MVPA or SB were not significantly different. In addition, an inverse association was observed between the number of components met and the depressive status (*P* for the trend = 0.006). The multivariable ORs were 0.40 (95% CI: 0.17, 0.92) for one component met, 0.34 (95% CI: 0.15, 0.75) for two components met, and 0.24 (95% CI: 0.10, 0.61) for all components met, compared with the “none of the components met” as a reference.

**TABLE 3:** The prevalence of depressive status (the Centers for Epidemiologic Studies Depression Scale ≥16) according to meeting 24-h movement guidelines (Setagaya- Aoba study, Japan, 2020).

	Number of participants	Number of cases	Crude OR	Multivariable OR^a^
Meeting recommendations				
None	38	12	1.00 (References)	1.00 (References)
SB only	58	6	0.25 (0.08, 0.74)	0.33 (0.09, 1.24)
MVPA only	79	16	0.55 (0.23, 1.32)	0.52 (0.15, 1.80)
Sleep duration only	29	5	0.45 (0.14, 1.47)	0.93 (0.22, 3.88)
SB and MVPA	190	23	0.30 (0.13, 0.67)	0.19 (0.06, 0.58)
SB and sleep duration	37	6	0.42 (0.14, 1.27)	0.44 (0.11, 1.74)
MVPA and sleep duration	66	9	0.34 (0.13, 0.91)	0.42 (0.11, 1.60)
SB, MVPA, and sleep duration	143	13	0.22 (0.09, 0.52)	0.22 (0.07, 0.70)
Number of meeting recommendations				
None	38	12	1.00 (References)	1.00 (References)
1	166	27	0.42 (0.19, 0.94)	0.50 (0.18, 1.42)
2	293	38	0.32 (0.15, 0.69)	0.26 (0.09, 0.72)
3^b^	143	13	0.22 (0.09, 0.53)	0.22 (0.07, 0.71)
*p* for trend			0.001	0.004

^a^ adjusted for age, sex, smoking status, drinking status, body mass index, changes in income, living alone, using social networking serviceservice service services, history of depression, number of medicated chronic diseases, and menopause (women only).

^b^ The number of meeting 3 recommendations meaning meeting SB, MVPA, and sleep duration: repost from mentioned above.

SB, sedentary behavior; MVPA, moderate-to-vigorous physical activity.

## Discussion

This study investigated the association between adherence to 24-h movement guidelines and the prevalence of depressive status using a survey conducted between Japan’s first and second states of emergency. Our primary findings were as follows. First, those who met the 24-h movement guidelines had a lesser depressive status than those who did not. Second, there was a strong association between SB and MVPA with a less depressive status. These findings emphasized the importance of meeting the guidelines, especially for adults. Thus, SB and PA were more important preventive components of a depressive status during the COVID-19 outbreak. However, the causal relationship remains unclear. Therefore, further longitudinal studies are required.

The prevalence of depressive symptoms during the lockdown was 27.8% in the United States and 24%–31% in China; WHO has reported an increase in the global prevalence of depressive symptoms to 25% due to the COVID-19 pandemic [32–34]. This study’s depressive symptoms prevalence was 14.1%, which was lower than the values mentioned above. It is unclear whether this is because the participants were not subjected to firm behavioral restrictions such as a lockdown. Yamamoto et al. conducted a nationwide online survey in Japan and found depressive symptoms prevalence of 17.9%, which is consistent with the present study [35].

Complete adherence to the recommended 24-h movement guidelines may improve health outcomes rather than independently meeting each component’s guidelines. Numerous previous investigations have shown an association between each component and depressive status. In addition, MVPA may improve health outcomes such as cardiovascular disease, biomarkers, and all-cause mortality [20]. However, to our knowledge, only a few studies have evaluated the association between adherence to the 24-h movement guidelines and mental health, including depression, for adults. Therefore, we believe this study is the first evidence of the benefit of 24-h movement guidelines for mental health. For those who stay at home due to the COVID-19 pandemic, reducing sedentary time to MVPA may improve or maintain mental health.

Those who decrease SB alone or increase MVPA, which meets the recommendation independently, may not be protected sufficiently from the onset of depression. Our study showed that those who met the recommendation in combination with SB and MVPA had less depressive status than those who met the recommendation for sleep duration alone or combined with sleep duration and MVPA or SB. The detailed mechanisms of this association are still unknown. However, the association of each component with depression is well established. Previous studies have reported dose-response relationships between MVPA, a lower risk of depression or SB, and a high risk of depression, regardless of the COVID-19 pandemic [5, 15, 16]. Additionally, a substantial health benefit may be obtained through SB reallocated to MVPA, not sleep duration [20]. Furthermore, there was a linear association between a less depressive status and meeting the recommendations of the Canadian 24-h movement guidelines. Accordingly, our study showed that there may have been an additive association between meeting the SB recommendations and the PA recommendations for reducing depression.

Plausible mechanisms of these findings may exist in multiple cascades. One possible mechanism is the biological aspect. Those with depression are often observed as having decreased hippocampal volume and increased oxidative marker levels [36]. PA can regulate the oxidant marker and hippocampal volume through activated regulation of brain-derived neurotrophic factors [37, 38]. A meta-analysis reported that a PA intervention might improve self-worth [39]. Additionally, increasing PA and subsequent cardiorespiratory fitness benefits cognitive function [40]. Finally, PA may improve mental health through an intricate interaction with each mechanism, while increasing SB may displace PA. Heavy SB decreases social interaction, which may increase the risk of depression. During the COVID-19 pandemic, increasing SB due to the increased time spent staying at home or in quarantine may have resulted in less time for face-to-face interactions when working and more time spent communicating remotely. Researching how to improve and prevent depression is necessary.

This study had several strengths. First, the survey was conducted during the COVID-19 outbreak, which caused a crucial health burden for many people. Although studies worldwide have reported on the association between PA and mental health during the COVID-19 pandemic [16], corresponding evidence among the Japanese population is limited. Moreover, few previous studies have adjusted for social factors to investigate the association between PA and mental health. Therefore, this study contributed substantially to the current literature. Second, PA was evaluated using a well-validated self-reporting questionnaire (GPAQ) [25]. A systematic review demonstrated the association of PA with depression during COVID-19 but did not include an appropriate PA assessment. Finally, to our knowledge, this study may have been one of few studies in adults and older adults to assess compliance with 24-h movement guidelines. This observation will provide essential knowledge to prepare for the next unknown infectious disease or disaster.

### Limitations

However, there were also several limitations to our study. First, the design of this study was cross-sectional; therefore, we could not determine a causal relationship between adherence to the 24-h movement guidelines and depressive status. Second, PA, SB, and sleep duration were self-reported. Although the GPAQ is a well-established and valid version of questionnaires, such as the International Physical Activity Questionnaire, the responses are subjective. There may have been recall bias. Objective measurements of PA, SB, and sleep duration are needed to exclude this bias. Third, the small sample size and restricted population were considered possible limitations. Fourth, habitual dietary information was not collected. Since it may play a role in the residual bias for the prevalence of depressive status, nutritional intake data should also be collected in future longitudinal studies [41]. Fifth, reverse causality may have existed when considering the associations between depressive status and PA or sleep duration [42]. A longitudinal design to investigate the onset of depression is needed in the future. Finally, the 24-h movement guidelines consensus panel removed the ≥10-min bouts of PA recommendation because accumulated <10-min bouts can promote health benefits [17]. However, the GPAQ asked participants to consider ≥10-min bouts of every PA domain; there was an inconsistency between the questionnaire and the guidelines. Thus, this inconsistency may have underestimated the PA. This discrepancy should be removed using an objective tool such as a 3-axis accelerometer.

### Conclusion

Meeting all recommendations of the 24-h movement guidelines, or at least meeting SB and MVPA recommendations, was associated with a lower prevalence of depressive status. This finding suggested that adults meet the 24-h movement guidelines to maintain a healthy mental status. Evaluating human life across 24 h is closer to the reality of life than evaluating each element in isolation. In addition, PA, SB, and sleep duration may be critical factors to consider in preventing depressive status during the COVID-19 outbreak, which could be useful knowledge for the next unknown pandemic crises. Nevertheless, further longitudinal and interventional studies are needed to confirm the effectiveness of the 24-h movement guidelines.
